# Primary Neuroendocrine Tumor of the Breast: Current Understanding and Future Perspectives

**DOI:** 10.3389/fonc.2022.848485

**Published:** 2022-05-25

**Authors:** Hongna Sun, Shuang Dai, Junnan Xu, Linan Liu, Jiaxing Yu, Tao Sun

**Affiliations:** ^1^ Department of Medical Oncology, Cancer Hospital of China Medical University, Liaoning Cancer Hospital & Institute, Shenyang, China; ^2^ Department of Medical Oncology, Lung Cancer Center, West China Hospital, Sichuan University, Chengdu, China; ^3^ Department of Pathology, Cancer Hospital of China Medical University, Liaoning Cancer Hospital and Institute, Shenyang, Liaoning, China

**Keywords:** primary neuroendocrine carcinoma of the breast, neuroendocrine neoplasia, clinicopathologic characteristics, diagnosis, treatment, prognosis, literature review

## Abstract

Primary neuroendocrine carcinoma of the breast (NECB) is characterized with heterogeneity, rarity, and poor differentiation, which is probably an underestimated subtype of breast cancer, including small cell NECs and large cell NECs. The diagnostic criteria for NECB have been constantly updated as the disease changes and the understanding increases. According to the latest WHO Classification, primary neuroendocrine neoplasm (NEN) of the breast consists of well-differentiated neuroendocrine tumors (NET), extremely aggressive neuroendocrine carcinomas (NEC) as well as invasive breast cancers of no special type (IBCs-NST) with neuroendocrine differentiation. The accurate diagnosis of NECB remains a challenge for its low incidence, which needs multi-disciplinary methods. For the rarity of the disease, there is a lack of large samples and prospective clinical research. For these invasive tumors, there are no standardized therapeutic guidelines or norms, and the treatment often refers to nonspecific breast cancer. In addition, the prognosis of such patients remains unknown. In 2003, the World Health Organization (WHO) listed NECB as an independent entity for the first time, while few features of NECB were clarified. In this review, it presents the WHO Classification, clinicopathologic characteristics, diagnosis, treatment, and prognosis of these patients. In addition, it summarizes the latest studies on molecular features of NECB, aiming to provide new therapeutic perspectives for the disease.

## Introduction

Neuroendocrine neoplasm (NEN) has features distinguished from other solid malignancies. Neuroendocrine cells scatter around the whole body with the dual characteristics of nerve cell-like structure and endocrine cell-like biological activity. As neuroendocrine cells are distributed throughout the whole body, NENs may appear in nearly all organ systems. NEN frequently occurs in the gastroenteropancreatic system and the bronchopulmonary system (1), and rare primary tumors occur in the skin, thyroid gland, bladder, and larynx (2–4). Primary neuroendocrine carcinoma of the breast (NECB) has characteristics of heterogeneity, rarity, and poor differentiation, and it is an underestimated subtype of breast cancer. Previous studies have reported that the incidence of NECB is variable for the rarity of the disease and the developing diagnostic criteria. In 2003, the World Health Organization (WHO) officially defined NECB as expressing neuroendocrine markers in more than 50% of tumor cells. The WHO Classification of tumors of the breast in 2012 objected to the edition in 2003 and suggested that the diagnosis could be confirmed regardless of the percentage (50% threshold) of tumor cells expressing neuroendocrine biomarkers (5). The latest WHO Classification 2019 unified NEN of the breast with that of other organ systems based on histological features and defined NEN into well-differentiated neuroendocrine tumors (NETs), highly aggressive neuroendocrine carcinomas (NECs), and invasive breast cancers of no special type (IBCs-NST) with neuroendocrine differentiation (6).

At present, there is no conclusion on the histogenesis of the disease. The main clinical features of NECB are breast lump, skin ulceration, bloody nipple discharge, and skin retraction, which are similar to those of IBC-NST. Compared to invasive ductal cancers of no special type (IDCs-NST), NECB is more likely to present systematic metastasis at diagnosis. In addition to clinical features, radiologic characteristics of NECB are nonspecific. Most NECB patients show positive estrogen receptor (ER) and/or progesterone receptor (PR) expression, implying that NECB is part of the luminal-like type (7). There are certain differences among NECB, IBC-NST, and IDC-NST in terms of morphological features and neuroendocrine biomarkers, which contribute to the diagnosis of NECB. Histogenesis and prognosis of NECB are still ill-defined. In addition, there are no standardized therapeutic guidelines or norms for these invasive tumors, and treatment often refers to nonspecific breast cancer reported in case reports and retrospective studies. Surgery remains the primary treatment for IDC-NST followed by taxane-based or anthracycline chemotherapy, endocrine therapy, and targeted therapy according to the receptor status. Given the low prevalence of NECB, knowledge is limited to case reports and small retrospective studies, and the understanding of the clinical features and management of this disease is limited. In this review, we summarize the clinicopathologic characteristics, diagnosis, treatment, and prognosis of these patients, and we elaborate on the molecular features of NECB to provide new therapeutic perspectives.

## WHO Classification

Neuroendocrine differentiation in breast cancer was first described in 1963 (8). In 1977, Cubilla and Woodruff presented a few breast cancer cases with a carcinoid growth pattern and produced the term breast primary carcinoid tumor (9). In 1985, Bussolati et al. demonstrated positive chromogranin A (CgA) expression in the normal mammary parenchyma, offering definitive proof of neuroendocrine (NE) differentiation (10). In 2000, Sapino et al. first proposed the diagnostic criteria for NECB, and they considered breast carcinomas resembling neuroendocrine tumors of the gastrointestinal tract and lungs in morphological features, demonstrating significant expression of neuroendocrine markers [greater than 50%, particularly CgA and synaptophysin (Syn)] (11).

Until 2003, the WHO Classification (the Third Edition) recognized that breast NETs were an independent breast entity (**Table 1**), and NECB was defined by morphological neuroendocrine features similar to those of gastrointestinal/pulmonary NETs. NETs of the breast were defined as tumors of epithelial origin with neuroendocrine marker (CgA and/or Syn) expression in more than 50% of tumor cells. These cancers were classified as large cell carcinomas, small cell/oat cell carcinomas, and solid NECs based on morphological features.

**Table 1:** Summary of different WHO classifications.

WHO	Terminology	Diagnosis	Subgroups
2003	Neuroendocrine tumor	**•** Morphological features similar to those of NE tumors of both GI tract and lung **•** Tumors of epithelial origin **•** Expression of neuroendocrine markers in more than 50% of tumor cells	**•** Large cell carcinomas **•** Small cell/oat cell carcinomas **•** Solid NE carcinomas
2012	Carcinomas with neuroendocrine features	**•** Morphological features similar to those of NE tumors of both GI tract and lung **•** Express NE markers regardless of the percentage (50% threshold) of tumor cells **•** Include IBCs-NST and special subtypes with NE differentiation	**•** NET, well differentiated **•** NEC, poorly differentiated/small cell carcinoma **•** IBCs with NE differentiation
2019	Neuroendocrine neoplasm	**•**>90% NE histological features or NE marker expression **•** Exclude solid papillary carcinoma and hypercellular subtype of mucinous carcinoma	**•** NET, well differentiated **•** NEC, poorly differentiated (small cell NECs; large cell NECs)
	IBCs-NST with neuroendocrine features	**•**≤90% NE histological features or NE marker expression 10-90%: mixed invasive (NST or other special type) and NECs <10%: invasive NST or other special types commented on the focal NE pattern	

In 2012, the WHO Classification objected to the edition in 2003, indicating that diagnosis could be confirmed regardless of the percentage (50% threshold) of tumor cells expressing neuroendocrine biomarkers (**Table 1**). NECB was regarded as “carcinomas with neuroendocrine features”, which was defined by morphological traits resembling gastrointestinal/pulmonary NETs. NENs were classified into three subgroups as follows: well-differentiated NETs (NETs), poorly differentiated/small cell carcinomas (NECs), and invasive carcinomas with neuroendocrine differentiation. The third group included IBC-NST and special subtypes with neuroendocrine differentiation (solid papillary carcinoma and mucinous carcinoma with neuroendocrine differentiation). However, this edition had small cell NEC but not large cell. The NET and NEC groups presented similar morphological traits as their gastrointestinal/pulmonary counterparts (5).

In 2019, the WHO Classification in the Fifth Edition (**Table 1**) unified NECB with NEN of other organ systems based on histological features to decrease confusion and inconsistencies in classifications, terminology, histologic grading criteria, and TNM staging. Within this framework, the terminology neuroendocrine neoplasms was introduced, including tumors with prominent neuroendocrine differentiation (presence of histologic neuroendocrine features in more than 90% of the tumor cells), and NEN was defined as NET when well differentiated and NEC when poorly differentiated. NEC was further divided into small-cell NECs and large-cell NECs. Furthermore, solid papillary carcinoma and the hypercellular subtype of mucinous carcinoma were excluded. Breast NETs were graded on the basis of the Nottingham grading system, which comprehensively evaluates the proportion of glandular tube formation, nuclear pleomorphism, and mitotic count in invasive breasts, with the quantity of mitoses continuing to be the main parameter in grading systems (6). In addition, if neuroendocrine biomarker expression or histological features make up ≤90% of the tumor area, it is defined as an IBC-NST with neuroendocrine features. When cancers have a 10–90% NEN pattern, the terminology of mixed invasive (NST or other special type) or NEC may be used, and the NEC percentage should be reported. Cancers with <10% NEN pattern should be classified as NST or other special types with an option to describe the focal specialized neuroendocrine pattern in the report comment.

## Epidemiology and Clinical Features

The changes in classification and different morphological and immunohistochemical criteria for the diagnosis of NECB from 2003 to 2019 result in a lack of uniformity in the terminology and definition of NECs, thereby hindering an accurate assessment of the incidence of NECB. Accordingly, the reported morbidity is extremely variable, ranging from 0.1% to 19.5% (12, 13). Wang et al. analyzed 381,644 cases of breast cancer from the database of surveillance, epidemiology, and end results (SEER). The results showed that according to the WHO diagnostic criteria of 2003, only 0.1% of breast cancer is NECB, which is lower than the 2–5% reported by the WHO in 2012 (5), suggesting that NECB may be underestimated because immunohistochemical (IHC) examination for neuroendocrine biomarkers is not routinely performed and cytomorphologic evaluation underestimates neuroendocrine differentiation. Therefore, it is difficult to confirm the true incidence of NECB (14).

NECB is a particular histologic subtype of breast cancer with similar morphological characteristics to gastrointestinal/lung NETs while displaying some degree of heterogeneity, including certain features that are usually difficult to identify from IBC-NST. Therefore, NECB may be misdiagnosed as metastatic breast cancer, carcinomas of IDC-NST, or breast carcinoma with neuroendocrine differentiation.

As the incidence of NECB is low, there is limited knowledge about the specific clinical characteristics of NECB. Most of the data comes from case reports and retrospective studies. The clinical feature of NECB is mainly characterized by a solitary breast lump, probably accompanied by skin ulceration, bloody nipple discharge, skin retraction, palpable axillary mass, and breast discomfort (15). Some people may have complications such as bone pain, respiratory symptoms, abnormal liver function, hematuria, and neuralgia caused by metastasis. While some people have no symptoms, they occasionally discover the disease due to routine mammographic screening. A few patients may suffer from carcinoid syndrome or hormonal hypersecretion. Patient age at diagnosis is mainly between the fifth and seventh decade of life (majority aged >60 years), ranging from 26 to 99 years, and most patients are postmenopausal women with higher clinical stage and histologic grade. However, few NECB patients are men (12, 15, 16). In addition, a previous study has reported one 13-year-old NECB patient (17). Several patients have a history of contralateral or ipsilateral invasive carcinoma of no special type with a tumor size ranging from 0.6 to 18.0 cm (mean: 2.3–3.7 cm), and approximately 40% of NECB has axillary lymph nodal metastasis at diagnosis (15, 16, 18, 19). Compared to IDC-NST, NECB patients are more likely to present systematic metastasis at initial diagnosis, and the most common metastatic sites are bone, liver, lungs, brain, bone marrow, and pleura, and several cases involve skin (18, 20–22). In addition, Kawasaki et al. reported peculiar endovascular spread (23).

There is limited understanding of the radiological features of NECB, and its radiological characteristics are nonspecific. Some studies have reported that NECB often exhibits the following characteristics: as a round, oval, or lobular mass with nonspiculated margins; a sharply circumscribed high-density mass on mammography; a hypoechoic solid mass with indistinct margins, which increases vascularity; and no enhanced posterior echo or a cystic component on breast sonograms (15, 24). Calcifications in NECB are uncommon in comparison with occurrence in invasive breast cancer (15). Magnetic resonance imaging has suggested an irregular mass with ill-defined margins, washout kinetics, and a marginal or heterogeneous internal enhancement pattern (15). In addition, PET-CT with 68 gallium-labelled somatostatin analogues can be used in well-differentiated NECB. 18-Fluorodeoxyglucose (FDG) PET-CT can be performed in poorly differentiated NECB or small cell carcinomas with high metabolic activity (25, 26). It is imperative to differentiate primary NECB from metastatic disease to the breast because it is common for metastatic neuroendocrine tumors to occur from other sites to the breast. Metastasis from other primary sites to the breast can be excluded by suitable methods, such as chest, abdominal, and pelvic computed tomography scans.

The diagnosis of NECB is based on morphological features and neuroendocrine biomarkers, and a biopsy is necessary for a definite diagnosis. Fine-needle aspiration (FNA) cytology may be inadequate for the diagnosis of NECB as the cytological features of NECB are parallel to those of intraductal papilloma and IDCs. Furthermore, the findings of FNA can be misinterpreted as adenocarcinoma (24). Therefore, the diagnosis is established by imaging-guided (ultrasound, stereotactic, or MRI guidance) core needle biopsy or specimens after surgery. Differential diagnoses include but are not limited to neuroendocrine tumors metastatic to the breast, lymphoma, Merkel cell carcinoma, and melanoma (27). The latest WHO diagnostic criteria for NECB stress the obligation to exclude the probability of metastatic neuroendocrine tumors from other organ systems because ≥97% of all neuroendocrine carcinomas originate from the gastrointestinal tract or lungs (1). It is not easy to differentiate these tumors in some situations, but the appearance of an associated ductal carcinoma component detected by histology is effective evidence of primary NECB (6, 28, 29).

## Histogenesis and Histopathology

At present, the histogenesis of NECB is still unclear. Some investigators have proposed that NECB does not originate from pre-existing and/or hyperplastic neuroendocrine cells but instead originates from differentiation events in breast cancer because they could not detect neuroendocrine cells in breast tissues (30). In contrast, Tomonori et al. demonstrated that benign neuroendocrine cells appear in the background of breast parenchyma with NECB and that they are arranged in isolated/scattered, clustered, and circumferential patterns, implying that neuroendocrine cell proliferation may be related to a precancerous state in the histogenesis of NECB (31).

**Figure 1:**
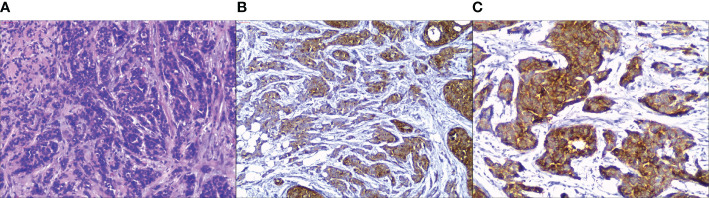
Pathological findings in primary neuroendocrine carcinoma of the breast. **(A)** H&E stain, tumor cells are composed of dense cellular solid nests, some of which are arranged in alveolar, with round or short spindle cells and eosinophilic cytoplasm. **(B)** Tumor cells show different degrees of positive expression for Chromogranin A on immunohistochemistry. **(C)** Tumor cells show strong and diffuse synaptophysin expression. (Hematoxylin-eosin, original magnification ×100 **(A)**; original magnification ×100 **(B, C)**.

NECB has clinical and radiologic characteristics that are difficult to distinguish from common types of breast cancers. Only approximately 33% of NECB patients can be diagnosed by morphology (32, 33). Consequently, the diagnosis of NECB is made by histology and IHC staining of neuroendocrine markers (**Figure 1**), which plays a critical role in improving the diagnostic rate of NECB. NECB is supported by the appearance of neuro-secretory granules and diffuse (more than 50%), uniform immunoreactivity for neuroendocrine biomarkers. Generally, NECB lesions vary from infiltrative mass lesions to well-circumscribed nodules, and some may have a focal hemorrhage with most tan and firm tumors (32). Histologically, low- or intermediate-grade invasive primary breast NETs are morphologically indistinguishable from their counterparts in the pulmonary region. Morphologically, the WHO definition indicates that breast NET consists of dense cellular solid nests and/or trabeculae of tumor cells in spindled, plasmacytoid, and polygonal shapes with eosinophilic and granular or clear cytoplasm separated by delicate fibrovascular stroma, rosettes, and peripheral palisading (6, 32). Although these tumors have similar cytological features, they may have an *in-situ* component to indicate a mammary gland origin. High Nottingham histologic grade NECB includes small cell NEC and large cell NEC. Small cell NEC accounts for approximately 0.1% of all breast cancers and 3–10% of extrapulmonary small cell carcinomas. Small cell NEC may be caused by the specific differentiation line of mammary cancer stem cells toward the neuroendocrine/small cell type, which can occur at the *in-situ* stage or later (at the invasive stage), rather than the malignant transformation of specific neuroendocrine cells in the normal breast tissue. Small cell NEC shows an infiltrative growth pattern and is composed of densely packed, reasonably uniform, small, dark hyperchromatic nuclei with a high N:C ratio, nuclear molding, scant cytoplasm, inconspicuous nucleoli, and poorly defined cytoplasmic boundaries (6). Similar to breast NETs, histologic and IHC profiles are challenging to distinguish from their lung counterparts. Thus, the appearance of ductal carcinoma *in situ* (DCIS) and the lack of tumors in other organs on radiologic imaging play vital roles in confirming the diagnosis of small cell NEC as a primary breast tumor. Large cell NEC is an exceedingly rare subtype of NECB. Large cell NEC presents highly pleomorphic nuclei with coarse chromatin and moderate cytoplasm.

The advent of the IHC technique makes it possible to identify the neuroendocrine phenotypes in breast cancer subpopulations by displaying their immunoreactivity to CgA, Syn, neuron-specific enolase (NSE), and CD56, which are usually negative in IBC-NSTs. CgA and Syn are the most sensitive neuroendocrine markers, whereas NSE and CD56 have lower sensitivity and specificity. In addition, some new second-generation neuroendocrine biomarkers, namely, INSM transcriptional repressor 1 (INSM1), ISL LIM homeobox 1 (ISL1) and secretagogin (SECG), have been introduced in clinical practice. Juhlin et al. compared these three new biomarkers with CgA and Syn, and they found that ISL1, INSM1, and SECG show the same or slightly lower sensitivity as CgA and Syn, confirming that the second-generation neuroendocrine markers present tissue-specific patterns, which are helpful to identify the primary tumor in the analysis of metastasis (34).

As described above, the essential differential diagnosis of NECB is a metastatic NET from the extramammary site. Because NECB and metastatic NEC to the breast show substantial morphologic overlap, differentiation is difficult. Some site-specific lineage markers can help distinguish NECB and metastatic NET, such as GATA3, mammaglobin, GCDFP15, TTF1, CDX2, and PAX8/PAX6. The most specific biomarkers for primary breast tumors are GATA3, mammaglobin, and GCDFP15, which are negative for secondary tumors (29). TTF1-, CDX2-, and PAX8/PAX6-positive cells are expressed at specific sites. Mohanty et al. reported that TTF1 is positive in approximately 70% of lung metastases, CDX2 is positive in 100% of gastrointestinal metastases, and CDX2 is consistently negative in NECB (29). PAX8/PAX6 positivity implies the origination of the gastric pancreas and duodenum. In addition, IHC staining for myoepithelial cells (such as smooth muscle myosin and p63) can aid in differentiating metastatic neuroendocrine neoplasms from *in-situ* carcinoma (29). Regarding the molecular subtype, most NECBs are hormone receptor-positive and human epidermal growth factor receptor 2 (HER-2)-negative, presenting a luminal-like phenotype, and ER may help distinguish the two entities (16, 35). However, ER positivity alone is not sufficient to determine the nature of the breast as it is neither universally expressed in common breast cancers nor specific to breast tumors. For example, metastatic NETs express ER in 13% (29) and 11% (36) of patients. In addition, some researchers have reported ER and PR positivity in pulmonary, pancreatic, small intestinal, and ovarian neuroendocrine tumors. Similarly, GATA3 has been reported in urothelial carcinomas and other tumors, including cutaneous squamous cell carcinomas, renal epithelial tumors, mesotheliomas, and autonomic nervous system tumors (37, 38). Furthermore, it should be noted that strong TTF1 positivity can also be observed in poorly differentiated mammary NETs (29). Either morphology or IHC markers of NECB have an overlap of metastatic tumors. Therefore, a relevant clinical history must be considered when making a definitive diagnosis.

## Molecular Features

There have been relatively few efforts to better characterize the mutational profile and molecular characteristics of NECB due to its rarity and heterogeneity. Here, we provide an overview of molecular alterations reported in NECB cases and provide a summary in **Table 2**. Evidence has demonstrated that NECB has different mutational profiles from other ER-positive and HER2-negative breast cancers with a lower frequency of PIK3CA mutations and a higher mutation rate in other genes. Ang et al. first reported a systematic investigation of activating mutations of NECB in 2014, identifying mutations in 5 of 15 (33%) NECs, including PIK3CA mutation in 20% of NECB and rare mutations in breast cancer (fibroblast growth factor receptor 1 (FGFR1), FGFR4, kinase insert domain receptor (KDR), and HRAS) (35). Caterina et al. showed that the most common mutated genes are GATA3, FOXA1, TBX3, and ARID1A (3/18, 17%; similar to lobular carcinomas), and they reported that there is a low frequency of PIK3CA, AKT1, and CDH1 mutations (2/18, 11%; identical to mucinous carcinomas) as well as no TP53 mutations (39). Although Vijayvergia et al. reported that TP53 is the most common mutation in poorly differentiated NECs, the locations of the primary site are almost always in the gastrointestinal system (41). Another study has shown that in contrast to standard forms of luminal cancers, NECB has a markedly low rate of PIK3CA mutations (7%) and TP53 mutations (7%) (18).

**Table 2:** List of molecular alterations in NECB.

Molecular alterations	Description	Ref.	Sample size
PIK3CA mutations	**•** Targeted sequencing analysis found three cases (7%) harboring PIK3CA mutations (18) **•** PIK3CA mutations in 20% of NECB and other rare mutations in breast cancer (FGFR1, FGFR4, KDR, HRAS) using a PCR/mass spectroscopy or semiconductor-based sequencing strategy (35) **•** The most frequently mutated genes were GATA3, FOXA1, TBX3, ARID1A (3/18, 17%), and PIK3CA, AKT1, CDH1 (2/18, 11%) (39).	Lavigne et al. (18) Ang et al. (35) Marchiò et al. (39)	42 15 18
TP53 mutations	**•** Targeted sequencing analysis found three cases (7%) harboring TP53 mutations (C277Y, Y220C, and H193R) (18). **•** No TP53 mutations were detected in NECB, enrichment for FOXA1, TBX3, ARID1A mutations (3/18, 17%), and PIK3CA, AKT1, CDH1 (2/18, 11%) (39).	Lavigne et al. (18) Marchiò et al. (39)	42 18
TROP-2, FOLR1, H3K36Me3	• TROP-2, FOLR1, and H3K36Me3 were three potential targets for novel therapies in NECB, CCND1, and FGFR gene amplification were found in isolated cases (40).	Vranic et al. (40)	20

Vranic et al. suggested several potential targets for novel therapies in NECB for the first time, including predicted expression of trophoblast cell-surface antigen 2 (TROP-2), folate receptor 1 (FOLR1), and H3K36Me3 in NECB, which may lead to the development of new targeted therapy drugs for NECB. In addition, these researchers found CCND1 and FGFR gene amplification in isolated cases. However, their study did not discover MGMT hypermethylation, DLL3 expression, or NTRK gene fusions. Furthermore, they reported that no biomarkers predict the efficacy of immune checkpoint inhibitors (programmed death-ligand one expression, microsatellite instability, and tumor mutational burden) (40). At present, all approved biomarkers that respond to PD-1/PD-L1 inhibitors have been demonstrated to be negative. The differences among studies are mainly due to the limited number of cases, the extent of genetic testing, and tumor heterogeneity. Although there are differences, these studies still provide helpful information about NECB and help us to find new targets for a more personalized therapy for this rare entity.

## Prognosis

Although the findings about the prognosis of NECB are controversial, most studies have shown that the prognosis of NECB is poor. In the prospective analysis of Rovera et al. (42), NECB had better survival than infiltrating ductal and lobular carcinoma. Nevertheless, Wang et al. (12) and Yang et al. (22) reported opposite results, showing that NECB had worse overall survival (OS) and disease-specific survival (DSS) than IDC-NST. Furthermore, there are similar outcomes in DSS and OS between large cell and small cell NECs. Another study has suggested that patients with NECB have shorter disease-free survival (DFS) than those diagnosed with IDC-NST, but no significant differences were observed in OS (18). When small cell NEC is specific to histologic subsets, it has the worst prognosis.

Previous studies on the prognostic significance of neuroendocrine differentiation in NECB have yielded contrary results due to different diagnostic criteria and the limited number of cases. Lai et al. (14) found that stratification based on the expression level of neuroendocrine biomarkers may provide information related to prognosis, which is conducive to exploring better treatment strategies; they found that NECB tends to be a luminal-like type. Patients with high expression levels of neuroendocrine markers are associated with less invasive clinical parameters (lower histologic grade, less lymph node metastasis, and lower stages), and the prognosis of these patients is better than those with regular expression levels (16). However, some studies have shown that patients with focal neuroendocrine differentiation have worse OS and DFS than those without neuroendocrine differentiation. Giuseppe et al. (43) reported that neuroendocrine differentiation is significantly associated with T4 stage, G2 grade, ER positivity, and PR positivity. Nevertheless, neuroendocrine differentiation does not affect breast cancer prognosis regarding breast cancer-specific survival (33).

In addition, cancer antigen 15-3 has been shown to be remarkably elevated in a patient at baseline and to significantly decrease after treatment, indicating that CA15-3 may be a prognostic factor (44). In most studies, patients with a large tumor size (>20 mm), higher stage, Ki67 > 14%, and hormone receptor-negative status are related to shorter OS (22). When referring to the influences of therapy strategy on the prognosis in NECB, patients who do not have surgery have poor DSS and OS, while those who receive chemotherapy have better DSS and OS in NENs. Wei et al. suggested that compared to conventional chemotherapy, endocrine treatment and radiation treatment show tendency toward survival benefit. However, none of the treatments reached statistical significance in their study, mainly due to the limited number of cases and short-term follow-up (16).

## Therapy

Multiple studies have shown that compared to IDC-NST, NECB is related to more invasive behavior and has a higher tendency for distant metastasis and local recurrence as well as a worse prognosis. However, given the rarity of NECB, there are currently no randomized controlled trials to compare treatment modalities or combinations of modalities in patients with NECB. Numerous treatments of NECB refer to the norm of ductal carcinoma reported in case reports and retrospective studies with surgery as the first-line therapy followed by taxane-based and/or anthracycline-chemotherapy, endocrine therapy, and targeted therapy according to the receptor status.

Surgery remains an essential method of treatment for early-stage NECB. The selection of surgery method for NECB resembles that for general breast cancer. Surgeons need to consider comprehensive factors, such as age, physical condition, tumor size and location, as well as the ratio of tumor size to breast volume. Of these factors, the size and location of the tumor determine the methods of surgery. There are many available surgical options, including breast-conserving surgery, modified radical mastectomy, breast reconstruction, and total mastectomy.

To date, there is still a lack of evidence for selecting the most effective chemotherapy protocols. Chemotherapy agents can be selected based on the histopathological characteristics of NECB. In general, poorly differentiated, small cell NEC or large cell NEC are treated with platinum/etoposide-containing regimens (45, 46). Taxane-based and/or anthracycline chemotherapy is used for other types of NECB (47). There is little evidence on whether NECB should be treated with neoadjuvant chemotherapy. Sanguinetti et al. treated a solid NECB using neoadjuvant chemotherapy with carboplatin and etoposide, which achieved a stable condition (48). Wei et al. reported that an NECB patient had a significant response after receiving four cycles of TEC (docetaxel, epirubicin, and cyclophosphamide) chemotherapy, resulting in a significant decrease in the Ki-67 proliferation rate from 40% to 10% (47). However, a conclusive recommendation cannot be suggested due to a limited scope of knowledge. Nonetheless, we suggest that patients with a large mass (>5 cm) with a powerful desire to preserve the breast, locally advanced NECB, or inoperable NECB can receive neoadjuvant chemotherapy. Adjuvant chemotherapy should be individualized, taking the biological characteristics and the risk of recurrence of the disease into account. High tumor grade, large tumor size, and lymph node metastases are essential negative prognostic factors for NECB (16).

As described above, studies have shown that the ER and PR in NECB are often highly expressed, presenting a luminal-like phenotype (16). Furthermore, endocrine therapy has a definitive effect on treating HR-positive breast cancer, indicating that it may be a helpful strategy in treating NECB. Some studies have reported that hormonal therapy combined with other therapy strategies is used to treat NECB when the tumor expresses the appropriate receptors (28). Zhang et al. showed that a young NECB patient who received goserelin and letrozole as neoadjuvant therapy achieved an excellent response (49). Neoadjuvant endocrine therapy can be used for patients with large tumors but who have a fervent desire to conserve the breast and who disagree with neoadjuvant chemotherapy. In addition, Shanks et al. presented the first patient with high-grade NECB who was resistant to platinum-based chemotherapy and hormone therapy but who obtained a remarkable response to palbociclib and the cyclin-dependent kinase (CDK) 4/6 inhibitor combined with fulvestrant (50).

HER2 positivity has been commonly related to poorly differentiated cancers of the breast. Anti-HER2 therapy can be used in sporadic cases of NECB, either in the adjuvant or metastatic setting with HER2 overexpression. Inga et al. reported a patient treated with anti-HER2 therapy in the adjuvant setting for HER-2-positive primary NECB who achieved 9-year DFS (51). Arpine treated a bone recurrent NECB patient with HER2 amplification who achieved a partial disease response after using trastuzumab (52).

Somatostatin analogues for the somatostatin receptor (SSTR) are targets for biological therapy in NETs. Somatostatin analogues show antiproliferative activity and prolonged PFS in small intestinal NETs (53). International guidelines recommend these analogues for the first-line treatment of well-differentiated G1/2 metastatic NETs. Liu et al. reported that a patient with NECB (large-cell NEC; Ki-67 proliferation index of 20%) and IDC received 177Lu-DOTATOC peptide receptor radionuclide treatment and achieved significant remission (44). Radiolabeled SSTR–targeted imaging and peptide receptor radionuclide therapy (PRRT) have demonstrated substantial benefit in managing SSTR-expressing NEN, revealing that PRRT may be a good choice for NECB (54).

In addition, NECBs may metastasize even years after treatment of the primary tumor. Therefore, long-term follow-up is recommended (55).

## Future Perspectives

Although there is no specific targeted therapy strategy for NECB, several new therapeutic medicines based on specific biomarkers have been investigated in other types of breast carcinoma and in NEC of the lung and gastrointestinal pancreas, which may provide a reference for treating NECB.

TP53 is frequently mutated in most human cancers, however, targeting TP53 mutation is difficult because of its structural diversity. Identifying a compound that can target all TP53 mutations is challenging. To date, there have been no approved targeted therapies for mutant TP53. Thus, instead of directly targeting TP53, exploiting mutant TP53 synthetic lethal genes and targeting noncoding RNA networks may provide additional therapeutic benefits (56). The mutation of PIK3CA has been observed in approximately 40% of patients with HR-positive and HER2-negative advanced breast cancer, which is much higher than in NECB patients (57). Everolimus inhibits mTOR through allosteric binding to mTORC (58). Based on the results of the BOLERO-2 trial, everolimus combined with the steroidal aromatase inhibitor exemestane has become standard therapy for patients with drug-resistant HR-positive and HER2-negative terminal breast cancer resistant to prior non-steroidal aromatase inhibitor therapy (59). Alpelisib is a PI3K inhibitor and degrader, and an oral biological preparation of alpelisib has demonstrated efficacy and a safety profile combined with fulvestrant in a phase 3 SOLAR-1 study for HR-positive and HER2-negative patients with PIK3CA mutations who were previously treated with aromatase inhibitors or CDK4/6 inhibitors (60, 61). Additionally, everolimus has been approved for lung, gastrointestinal, and pancreatic NETs. Thus, targeting PIK3CA in metastatic NECB may be a promising treatment strategy based on the efficacy and safety of alpelisib and everolimus in HR-positive and HER2-negative metastatic breast cancer.

TROP-2, a transmembrane glycoprotein, was initially discovered to be expressed at high levels on the surface of trophoblastic cells, affecting the growth, invasion, and metastasis of tumors. Most TROP-2 proteins are expressed in triple-negative breast cancer (TNBC), and Vranic et al. (40) detected TROP-2 proteins in 21% of patients, indicating that TROP-2 may be a potential therapeutic target for antibody–drug conjugates. The antibody–drug conjugate, sacituzumab govitecan, which targets TROP-2, has been shown to be highly effective in heavily pretreated patients with mTNBC with good toleration (62). Mammary gland FLOR1, which encodes a leucine-rich repeat protein and is mainly expressed in TNBC, is related to worse clinical outcomes and involves cancer cell signaling and growth, suggesting that it may be a promising target for treatment strategies, such as antibody–drug conjugates (63). Mirvetuximab soravtansine is an antibody–drug conjugate that is currently being assessed in multiple clinical trials (63, 64). Although TROP-2 and FLOR1 are mainly expressed in TNBC, the discovery of TROP-2 and FLOR1 in NECB suggests that neuroendocrine carcinoma and TNBC may share some elements of molecular pathogenesis, which may aid in the development of new targeted therapy drugs for NECB. As in the era of precision medicine, it is possible to achieve the same treatment of different diseases possessing the same gene mutation or protein expression, but further clinical trials are necessary.

FGFR signaling is often deregulated in various cancers, including breast cancer and even in some cases of NECB (35). The FGFR pathway plays an essential role in tumor growth and survival, providing a promising therapeutic option for developing FGFR inhibitors. The favorable clinical benefits observed in tumors have contributed to the approval of FGFR inhibitors, including pemigatinib and infigratinib, which have been approved for patients with FGFR2 fusion/rearrangement-positive cholangiocarcinoma (65), as well as erdafitinib, which has been approved for patients with FGFR-aberrant urothelial carcinoma (66). Thus, NECB patients with FGFR aberrations may benefit clinically from FGFR inhibitors.

Additionally, the activating mutation of KDR (VEGFR2) in some patients with NECB may provide the theoretical basis for the investigation of antiangiogenic agents in this disease. Pazopanib, an oral multitargeted tyrosine kinase inhibitor, acts through VEGFR types 1–3. A systematic review has elucidated the efficacy and safety of pazopanib in patients with locally advanced and metastatic NEN, indicating that pazopanib may be an option for NECB patients (67). Immunotherapy using checkpoint inhibitors that block PD-1/PD-L1 has emerged as a highly effective therapy in numerous patients across a range of malignancies. However, studies evaluating all currently approved biomarkers in response to PD-1/PD-L1 inhibitors have been demonstrated to be negative, indicating that NECB patients may not benefit from immunotherapy. However, PD-1/PD-L1 inhibitors have shown effectiveness on high-grade NENs from other sites with high PD-L1 expression or a high number of tumor-infiltrating lymphocytes (68).

## Conclusion

NECB is a rare neoplasm, and its biological behavior, clinical features, treatment, and prognosis are not yet fully understood. Although some NECB patients can benefit from conventional cytotoxic chemotherapy, others are resistant to chemotherapy. Improvements in understanding the molecular characteristics of NECB have led to the development of molecular targeted therapy for this group of diseases. In the era of precision medicine, priority should be given to identifying therapeutic targets, highlighting the role of molecular-driven studies on neuroendocrine malignancies, and modulating these targets with specific inhibitors, thus producing great clinical benefits.

## Author Contributions

HS and SD reviewed the literature and drafted the article. JX and JY conceived the review and drew the tables. LL provided the pathological figures of breast primary neuroendocrine tumor. TS revised the manuscript. All authors read and approved the final manuscript.

## Funding

This study was partly funded by Liaoning Province Key Laboratory Project of Breast Cancer Research (2016-26-1, TS), Shenyang Breast Cancer Clinical Medical Research Center (2020-48-3-1, TS), Medical-Engineering Cross Research Fund between Liaoning Cancer Hospital and Dalian University of Technology (LD202022, TS), “Metabolic Abnormality and Tumor” Research Project (ZP202017, TS), Beijing Medical Award Foundation (YXJL-2020-0941-0752, TS), and Wu Jieping Medical Foundation (320.6750.2020-12-21,320.6750.2020-6-30,320.6750.18541, TS).

## Conflict of Interest

The authors declare that the research was conducted in the absence of any commercial or financial relationships that could be construed as a potential conflict of interest.

## Publisher’s Note

All claims expressed in this article are solely those of the authors and do not necessarily represent those of their affiliated organizations, or those of the publisher, the editors and the reviewers. Any product that may be evaluated in this article, or claim that may be made by its manufacturer, is not guaranteed or endorsed by the publisher.
